# User expectations and perceptions towards new public transport infrastructure: evaluating a cable car in Bogotá

**DOI:** 10.1007/s11116-021-10260-x

**Published:** 2022-01-28

**Authors:** Luis A. Guzman, Victor A. Cantillo-Garcia, Julian Arellana, Olga L. Sarmiento

**Affiliations:** 1 Grupo de Sostenibilidad Urbana y Regional, SUR, Departamento de Ingeniería Civil y Ambiental, Universidad de los Andes, Bogotá, Colombia; 2 Department of Civil and Environmental Engineering, Universidad del Norte, Barranquilla, Colombia; 3 School of Medicine, Universidad de los Andes, Bogotá, Colombia

**Keywords:** Public transport, Cable car, Urban ropeways, Expectations, Perceptions, Discrete choice models, Bogotá

## Abstract

Cable cars are a viable alternative to improve citizens’ accessibility in zones with limitations on urban public transport supply due to the topography. In Latin America, such systems have recently been implemented in zones with high levels of poverty and vulnerability. Although the social implications of their implementation are relevant, individual expectations of these systems and how current changes in travel conditions and quality of life are perceived have not been widely reported in the literature. This paper aims to evaluate users’ expectations and perceptions of a new cable car in the southern periphery of Bogotá (Colombia). We conducted a panel survey before (n = 341) and after (n = 301) the cable car started operations to evaluate the ranking of preferences toward a set of possible benefits of the project. We estimated discrete choice models to analyze the statistical differences between the expectations and perceptions before and after changes. Results suggest that travel time reductions, comfort improvements, and in-vehicle security are the benefits most valued by the users. Even though the project meets expectations of these aspects, it seems to fall short in expectations of reductions of pollution. Individuals’ experience with the cable car shapes their perceptions of the system. We found that perceptions differ between those who have used the service at least once and those who never did. Policy implications derived from this study might be of interest to decision-makers seeking to guarantee the public acceptability of urban projects.

## Introduction

Cable car systems are an alternative to improve mobility and provide accessibility in urban areas with limitations on the supply of traditional public transport services. In the Latin American context, several projects have been implemented in the last two decades, usually in peripheral urban areas characterized by steep hills, narrow streets, informal settlements, poverty, and social exclusion. Moreover, cable cars might reduce commuting time and improve the accessibility of specific population segments with transport disadvantages (Garsous et al. 2019) while providing environmental and social benefits (Biberos-Bendezú and Vázquez-Rowe 2020; Sarmiento et al. 2020).

One of the main objectives of transport investments in urban areas is to improve the quality of life (QoL) of the population while meeting sustainable development goals (SDGs). To achieve this, some authors argue that it is necessary to update the traditional supply-orientated transport planning approach balancing the social dimensions and the physical components (Banister 2008). At this point, involving the community through their expectations and perceptions is a relevant aspect in the social dimension of the transport planning process, as they are relevant factors in the public acceptance of a project. The formulation of transport policies, including new infrastructure, must consider users’ expectations and perceptions that influence their decision to travel on public transport (dell’Olio et al. 2011).

In transport planning, decision-making without considering the target population’s needs and the built environment can lead to poor cost-effective projects that do not meet demand expectations and underestimate future planning procedures (Louw et al. 2013; Mackett and Edwards 1998) and ridership. This is in part due to differences in the perceptions of users and policymakers (Chowdhury et al. 2018). In fact, the evaluation of user perceptions allows designing policies that meet their specific expectations by identifying the factors that define the willingness to comply with planning outcomes (Beirão and Sarsfield Cabral 2007).

It seems necessary to understand the implications of people’s preferences and expectations of new public transport projects. As public acceptance and project appropriation drives political viability and funding, this is crucial to assure the execution and success of the interventions proposed by urban planners. This research aims to evaluate the expectations and perceptions of a new cable car (TransMiCable) in Bogotá, Colombia, and provide insight into new alternative public transport infrastructure in a context of social vulnerability that might be extrapolated to other developing cities. For this purpose, we conducted a panel ranking survey and relied on discrete choice modeling techniques to explore the differences between the users’ expectations and perceptions. It is worth noting that we refer to expectations (ex-ante) as users’ expected benefits of the cable car to be implemented. In contrast, perception (ex-post) refers to the opinion of users when TransMiCable is in operation.

To the best of our knowledge, this is the first study to compare the expectations regarding the changes in several attributes when implementing a cable car and perceptions after its implementation. The results of this paper might be useful to inform debates regarding real changes that citizens can expect to experience when a cable car is implemented, and the level of expectation fulfillment for the specific case of TransMiCable.

## Expectations and preferences in public transport

The literature addressing public transport and user perceptions are scarce. The analysis of user perceptions in public transport allows identifying the defining factors of travel demand, user satisfaction, and quality of service. Evidence shows that the main factors defining public transport usage by car users are primarily affective and associated with individual perceptions, motivations, and local context (Redman et al. 2013). Specifically, the literature confirms the potential of cable cars as an alternative to improve mobility in terrain constrained zones (Alshalalfah et al. 2012; Brand and Dávila 2011; Reichenbach and Puhe 2018; Tiessler et al. 2019), while other studies focus on the evaluation of benefits such as travel time savings (Garsous et al. 2019) and accessibility improvements (Heinrichs and Bernet 2014).

In addition, the comparison of perceptions and expectations is useful for policymakers to identify user preferences and factors that influence travel behavior and satisfaction. The perceptions of users and policymakers might not be aligned and understanding of this gap is limited. A case study in Auckland (NZ) evaluated the perceived importance of five attributes by policymakers and public transport users in the context of a new regional plan. There, Chowdhury et al. (2018) found that even though there were certain similarities, some differences were primarily related to the implications of transfer times, a more relevant attribute for frequent users. A joint analysis of expectations and perceptions could be a tool to account for user preferences in the planning process, strengthening social participation (Booth and Richardson 2001; Legacy 2017). Recent work in Indonesia addresses the links between road-based public transport users’ preferences and expectations. Main findings indicate that sociodemographic attributes and dissatisfaction correlate with the level of acceptance of a series of hypothetical public transport improvement measures (Joewono et al. 2016). Dahlan and Fraszczyk (2019) compared the expectations of a new mass public transport system among users that reside close to its catchment areas and citizens from other areas, finding some statistically significant differences between groups. Reliability and safety were more valued by the respondents from both groups when selecting a transport mode. It has been documented that if the quality of service of a bus system does not meet the users’ expected levels, the users migrate to other modes of transport, such as private vehicles and paratransit services (Deb and Ali Ahmed 2018).

Among other aspects, there is evidence of a strong positive correlation between the acceptance of transport pricing policies and the expectations of the users, which depend on individuals’ belief in the expected effects of the policies (Schuitema et al. 2010). Moreover, in the context of new transport infrastructure, the impacts expected and perceived by the community influence its engagement with the investment (Crane et al. 2016). 

Due to the nature of the variables involved in analyzing perceptions and expectations, some approaches rely on ordered regressions and discrete choice modeling. Dell’Olio et al. (2010) and Echaniz et al. (2018) estimated ordered probit regressions to model user perceptions of the quality of public bus services. Both studies conclude that driving style, reliability, waiting time, and travel time are the most valued attributes. These techniques are also applied to value the quality of service in public transport systems. They might be used to evaluate users’ perceived and desired (or expected) quality of service system (dell’Olio et al. 2011). In logistics, Amaya et al. (2020) estimated multinomial logit models with rank data to analyze the perceptions of carriers, receivers, and citizens concerning a set of urban freight policies in two Colombian cities. 

Applying an analytical hierarchic process (AHP), Jain et al. (2014) established a criterion ranking for shifting urban commuters to public transport in Delhi, indicating that safety, reliability, cost, and comfort make up the top four user-perceived attributes. AHP has also been used to model gaps in perception between current and potential bus users. For instance, current and potential bus users have different patterns of satisfaction levels and distinctive subjective evaluation of transport systems (Mahmoud and Hine 2013).

Rank data and best–worst models are alternative methods to evaluate and compare perceptions and attitudes that characterize qualitative and subjective valuations. With this type of data, best–worst approximations are used to model the trip experience on public transport systems (Beck and Rose 2016), to measure the satisfaction of public transport services (Echaniz et al. 2019), to identify barriers to walkability (Larranaga et al. 2019), to assess the relative importance of different dimensions and criteria in logistics (Badri Ahmadi et al. 2017; Rezaei et al. 2018), and parking choice (Orozco-Fontalvo et al. 2020). In addition, best–worst and rank data can be used to estimate consistent discrete choice models based on the random utility theory (Delle Site et al. 2019). Other approximations to evaluate factors influencing the perception of public transport service quality include classification trees (De Oña et al. 2012), principal component analyses (Nordfjærn et al. 2014), logistic regressions (Mahmoud and Hine 2016), linear regression (Wan et al. 2016), and structural equation modeling (Deb and Ali Ahmed 2018).

The review evidence that joint analysis of users’ expectations and perceptions might contribute to the transport planning process by identifying users’ specific needs and monitoring service quality and satisfaction. Furthermore, cable car services is a topic that stills has a margin for research.

## Urban transformations: the TransMiCable project

**Fig. 1:**
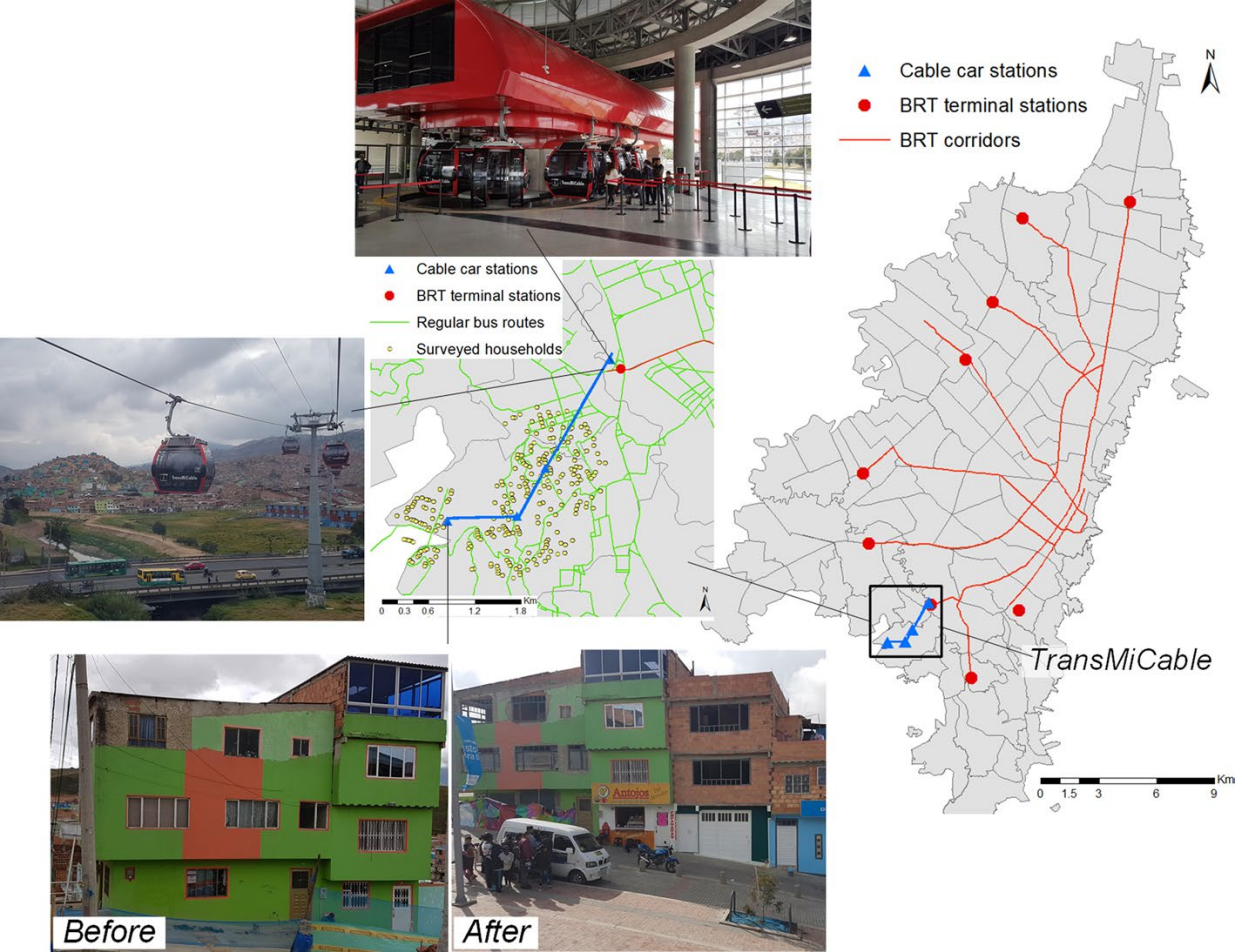
TransMiCable system

TransMiCable is a cable car integrated into Bogotá’s Public Transport System (SITP in Spanish). It started operations in December 2018 to provide better access to originally informal and poor settlements located in hillside areas of the south of Bogotá’s periphery. The project in operation consists of one line with four stations, with a total length of 3.43 km (Fig. 1). The travel time to complete the route is approximately 15 min, and the capacity is around 3600 passengers per hour. Before the COVID-19 pandemic, the cable ridership was around 22,000 passengers in a typical day. Currently, travel demand is 20,000 passengers per day.

The cable car is connected to a terminal station of the Bus Rapid Transit (BRT) system named *Portal Tunal*. Before the cable car, a typical peak hour journey between the *Portal Tunal* and *Mirador del Paraíso* (i.e., the highest cable station) could take up to one hour using a regular bus (with an integrated fare structure with the BRT) or paratransit services, so the cable car offers a saving in travel times of almost 80%. The project also includes a complementary urban redevelopment plan, including additional facilities for cultural, recreational, and social activities, community centers, and a program to support home improvements to reduce geomorphological hazards in the zone (Sarmiento et al. 2020). The TransMiCable has operative and fare integration with the BRT system. Transfers between these two services are possible at the *Portal Tunal* without additional charges.

In Bogotá, spatial economic segregation is evident, with lower-income households located mainly in the south and southwestern urban periphery, corresponding to the most densely populated and less accessible zones, while high-income households, the central business district, and most opportunities for work and study are found in the central and northeastern areas (Guzman et al. 2017; Guzman and Oviedo 2018). The population in the zone of influence of the TransMiCable represents a socially vulnerable population, with low income, unplanned urbanization, a high percentage of self-built informal urban settlements, and poor accessibility. According to our data, 94% of the households have an income lower than a monthly minimum wage (approximately 250 USD in 2019), while the mean household size is 3.8 residents. Moreover, around 96% of the households are cataloged at the lowest level of socioeconomic strata (SES), an official housing classification system with six levels according to physical characteristics usually associated with income, used primarily to focalize public service subsidies (Cantillo-García et al. 2019).

Before the inauguration of the cable car, the most frequently used modes of transport were regular bus and paratransit services provided by private vehicles. Mobility was characterized by high travel times due to the congestion generated by the interaction between traffic, the steep hills, and the circuitous streets that form the urban structure of the zone. Adding to the limited travel choices due to institutional constraints, such a situation can lead people to be socially excluded, having to expend valuable resources and time to fulfill their mobility requirements (Oviedo Hernandez and Titheridge 2016).

Given this background, the social impacts of the TransMiCable are relevant to meet the objective of improving citizens’ quality of life by improving accessibility and the provision of more and better communal spaces and services. In particular, analyzing the expectations and current perceptions of users can help to understand the real needs of the people and their evaluation of the impacts of the new service.

## Data and methodology

### Data collected

For the analysis, we rely on panel data collected in the influence area of the TransMiCable. The data collection had two phases. Firstly, a baseline face-to-face survey was conducted before implementing the project between February and November 2018; then we carried out a follow-up measurement from July 2019 to March 2020. The target population was adults living within 800 m of the TransMiCable stations for at least two years and with no plan to move out for at least two more years.

The overall sample of the study was selected through a multi-stage sampling design. Blocks were selected with a probability proportional to the density of parcels. Every third household was systematically selected. Lastly, we randomly selected one eligible adult per household. If the selected adult was not present or did not agree to participate the household was replaced until completing about 800 adults per area. This procedure allowed to complete a sample size powered to detect changes equivalent to standardized mean differences in outcomes that range between 0.3 and 0.4 (Sarmiento et al. 2020). Hence, the sample is statistically representative of the population of adults living in the influence area of the cable car.

At the baseline, we applied a general questionnaire of revealed preferences to 1031 individuals to gather sociodemographic, mobility, accessibility, and other health and social information. Among these persons, we randomly chose 343 participants to answer a stated preference experiment to assess their willingness to use the TransMiCable, a perceptual questionnaire about satisfaction with the current transport system, and a ranking survey to explore expectations of the cable car implementation. In the follow-up round, we contacted the same individuals to respond to a similar general questionnaire, a new stated preference exercise, and a ranking survey to explore perceptions of the TransMiCable. At this stage, 303 respondents (from the 343 selected) successfully answered all survey components, achieving a reasonable 11.6% attrition rate in the sample. A data cleaning process resulted in the exclusion of two observations for missing and incongruent information. Therefore, the final sample size consists of 341 observations for the baseline survey (i.e., expectations stage) and 301 for the follow-up round (i.e., perceptions of reality). Table 1 includes a description of the sample for both periods.

**Table 1:** Sample description

		Expectations (baseline)	Perceptions (follow-up)
	Sample Size	341	301
	Attribute	Proportion	
Time living in the house (years)	< 8	26%	22%
	8–25	39%	41%
	> 25	35%	37%
Age	18–28	21%	19%
	28–41	24%	23%
	41–58	26%	24%
	> 58	29%	35%
Sex	Female	73%	72%
	Male	27%	28%
Marital status	Single	20%	18%
	Married or domestic partner	53%	53%
	Divorced, separated, or widow	27%	29%
Education level	Primary	44%	45%
	Secondary	42%	42%
	Higher education	13%	14%
Occupation	Occupied: studies or works	59%	65%
	Non occupied	41%	35%
Vehicle ownership	Motorcycle	14%	20%
	Car	6%	5%
Household Income	< 250 USD/month	56%	49%
	250 – 500 USD/month	37%	42%
	> 500 USD/month	6%	9%
Socioeconomic strata	SES 1	96%	97%
	SES > 1	3%	3%
Owns the house where living		42%	43%
Household size < 4		39%	44%
Has ever used TransMiCable		–	89%
Uses TransMiCable regularly		–	16%

The sample is characterized by high unemployment, low education levels, and low household income. Most of the respondents were female; time living in the neighborhood has a mean of 19.3 years and a standard deviation of 13.2. Most of the individuals (85%) have used the cable car, while around 16% use it frequently for their daily trips. Many people do not use the cable car regularly because they carry out most of their daily activities inside the neighborhood.

### Ranking questionnaire

We issued ranking surveys before and after the TransMiCable went into operation. We asked respondents in both periods to rank the three attributes they expected to improve or perceived to be the most improved after the cable car started operating. We decided that individuals would rank only three attributes to minimize boredom and fatigue effects in the choice process that may affect the reliability of the data, considering that as the number of choices increases people tend to respond less carefully (Bradley and Daly 1994). Also, individuals usually classify the best and worst alternatives more easily, given that they have greater certainty about preferred and extreme alternatives. At the same time, they are less sure about middle options, which may be ranked with less care (Ortúzar and Willumsen 2011). The set of attributes from which to choose was the following.

- A1. Reduce travel time
- A2. Improve comfort
- A3. Improve reliability of waiting time
- A4. Improve in-vehicle security
- A5. Improve security at the station
- A6. Improve road safety
- A7. Increase the number of places I can access
- A8. Increase the number of hours (departure time) I can travel
- A9. Improve the frequency of the service
- A10. Reduce the fare
- A11. Reduce pollution
- A12. Improve reliability of arrival time
- A13. Improve the neighborhood aesthetic
- A14. Improve the quality of life
- A15. Nothing
- A16. Other

The database for our analysis consists of the sample who answered the ranking component in both surveys. It is noteworthy that along with alternative *A16* (Other), we encouraged respondents to specify which benefits, not in the list, they expected to improve after the cable car implementation. At the baseline, this allowed us to reclassify other responses into new alternatives. At the follow-up, we could not reclassify some answers because they referred to unique attributes, such as a better experience on the trip or better customer service offered by the cable operator, compared with informal paratransit services.

The use of rank data allows capturing more variability of the stated choices when compared to single-choice experiments. This reduces the bias related to the issue that all attributes might improve, and relative changes could influence the differences between expectations and perceptions compared to the top attributes that improved the most. Even when an attribute is not ranked within the highest set by an individual, the aggregation of responses from different individuals allows comparing aggregate relative weighting in a before and after situation, which can suggest if expectations were met.

The survey also asked the respondents to rank the three attributes they expected or perceived to get worse. These additional data were used to test the best–worst approaches. However, this was futile since most of the sample responded ‘nothing’, so the new data did not provide enough variability to enrich the models.

### Methods

The analysis reported in this paper is based on the modeling of the rank data collected. Rank data is a common source of information in quantitative research of many fields, such as psychology, sociology, and econometrics. Approaches to modeling rank data include order statistics, distance-based, decision trees, paired comparison, and multistage models. Probabilistic ordered models are the most popular approach due to their long history and wide use of literature on statistics and psychology (Yu 2000).

Discrete choice models allow estimating the probability of choosing an alternative from a set of available alternatives, measuring the effect of covariates, and capturing the heterogeneity of this probability. In particular, the Luce Model (Luce 1958), an extension of the multinomial logit model, estimates the choice probability from a set of stated choices in the form of ranks. Luce’s theorem supports this formulation, stating that a ranking can be decomposed into a sequence of *S–1* independent choice stages or pseudo-observations. Here, *S* refers to the number of alternatives in the ranking.

Based on this, we divided the rank into implicit choice observations. The first choice consists of the selection of the preferred alternative when all options are available. The second observation is the choice of the second-best alternative when the preferred attribute is not available. Finally, the third choice refers to the third-best alternative when the two preferred are not available. The choice probability is then given by Eq. 1.

1 $$\Pr \left( {r_{1} ,r_{2} ,r_{3} } \right) = \Pr \left( {{\raise0.7ex\hbox{${r_{1} }$} \!\mathord{\left/ {\vphantom {{r_{1} } {r_{1} ,r_{2} ,r_{3} , \ldots ,r_{j} }}}\right.\kern-\nulldelimiterspace} \!\lower0.7ex\hbox{${r_{1} ,r_{2} ,r_{3} , \ldots ,r_{j} }$}}} \right) \times \Pr \left( {{\raise0.7ex\hbox{${r_{2} }$} \!\mathord{\left/ {\vphantom {{r_{2} } {r_{2} ,r_{3} , \ldots ,r_{j} }}}\right.\kern-\nulldelimiterspace} \!\lower0.7ex\hbox{${r_{2} ,r_{3} , \ldots ,r_{j} }$}}} \right) \times \Pr \left( {{\raise0.7ex\hbox{${r_{3} }$} \!\mathord{\left/ {\vphantom {{r_{3} } {r_{3} , \ldots ,r_{j} }}}\right.\kern-\nulldelimiterspace} \!\lower0.7ex\hbox{${r_{3} , \ldots ,r_{j} }$}}} \right)$$

where *r*_*k*_ refers to the alternative ranked in position *k* (*k* = 1, 2, 3) and *Pr* (*r*_*1*_, *r*_*2*_, *r*_*3*_) is the probability of observing a given rank order considering the availability of *r*_*j*_ alternatives. This modeling framework is also called exploded logit (Ortúzar and Willumsen 2011).

In our specification, the probabilities in Eq. (1) follow the structure of a mixed logit model accounting for the panel effect due to the multiple observations by the respondent (Ortúzar and Willumsen 2011). Equation (2) describes the utility function *U*_*iq*_ associated with alternative *i* and individual *q*:

2 $$U_{iq} = { }ASC_{i} + { }X_{iq} \times \beta_{iq} + \varepsilon_{iq} + \eta_{q}$$

where *ASC*_*i*_ is the alternative specific constants, representing the net influence of all unobserved or not explicitly included characteristics of the individual and the alternative in the utility function (Ortúzar and Willumsen 2011); *X*_*iq*_ are observed attributes (e.g., socioeconomic characteristics); *β*_*iq*_ is a set of parameters to be estimated; *ε*_*iq*_ is a random error component with an identical and independent Type I Extreme value distribution; and *η*_*q*_ is a normal error component with mean zero and standard deviation to estimate, accounting for the panel effect. This error component varies across individuals but is constant over the repeated implicit observations of each individual according to the density function *f*(*θ*|*η*), conditioned to the population parameters *θ*. The unconditional probability is then given by Eq. (3), which can be estimated by applying simulated maximum likelihood methods (Train 2009):

3 $$P_{iq} = \smallint \mathop \prod \limits_{k} \frac{{e^{{U_{iq} }} }}{{\mathop \sum \nolimits_{j = 1}^{j} e^{{U_{jq} }} }}f(\theta |\eta )d\eta$$

We estimated two groups of models. Firstly, we estimated aggregated models to compare the overall expectations before the construction of the cable car versus perceptions after its implementation. In this case, we estimated market share models considering only *ASC*_*i*_ in the utility functions (*β*_*iq*_ = 0) assuming homogeneity in the perceptions. The second group of models considers *β*_*iq*_ parameters associated with the attributes sex, household income, occupation, and use of the TransMiCable. These models aim to capture perception heterogeneity according to these covariates. Both groups of models account for the panel effect through the inclusion of random error components *η*_*q*_.

The final number of pseudo-observations for the exploded models is 979 for the baseline (i.e., expectations) and 837 for the follow-up stage (i.e., perceptions). In some cases, the respondent did not report the complete ranking and only selected one or two alternatives. Furthermore, we evaluated the perception heterogeneity considering the catchment areas of the TransMiCable stations but did not find significant differences across these areas. We hypothesize that population characteristics are homogenous in the whole study area and there are no significant spatial differences in respondents’ perceptions.

## Results and discussion

**Fig. 2:**
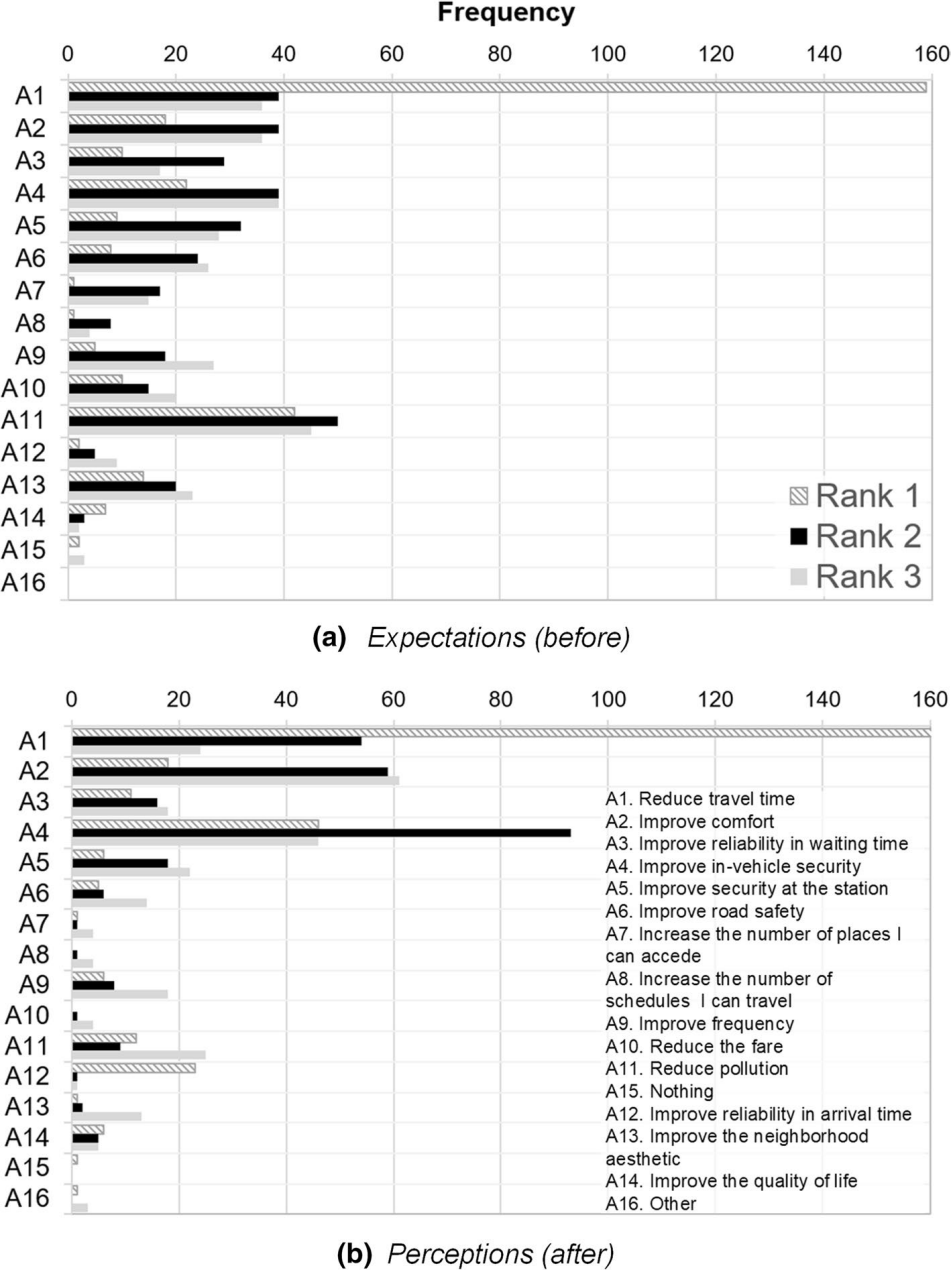
Ranking of alternatives: expectations (before) and perceptions (after)

Figure 2a displays the frequency with which each alternative was expected to be the first, second, and third attribute to improve the most after the TransMiCable implementation.

Figure 2b shows the current perceptions after implementation of the cable car, collected at the follow-up stage. Reductions in travel time dominate the ranking in both periods, but some differences emerge when comparing expectations and reality. Expectations of reduced pollution, security at the station, reliability, accessibility, and road safety seem to be higher than the benefits perceived. In contrast, perceptions of in-vehicle security and comfort exceeded expectations.

### First group of models estimation

Table 2 presents the results for the first group of models assuming homogeneity, including estimates for the alternative specific constants, t-test, the probabilities of being ranked as the most preferred alternative, and the respective rank according to these probabilities. Results are in line with the descriptive analysis of the rankings presented in Fig. 2. Reduction in travel time (A1) is the benefit expected as well as perceived to improve the most, positioned in the first place in both periods. Comfort (A2) and in-vehicle security (A4) are the benefits that the users perceived to be most improved compared to their expectations. Even though their ranking only changed one position, the probabilities were more than doubled at the follow-up.

**Table 2:** Aggregate model estimates

Attribute	Expectation				Perception			
	Estimate	t-test	Pi	Rank	Estimate	t-test	Pi	Rank
A1. Reduce travel time	4.725	10.41	55%	1	5.426	9.27	57%	1
A2. Improve comfort	2.479	6.83	6%	4	3.691	7.48	10%	3
A3. Improve reliability in waiting time	1.773	4.92	3%	8	2.326	4.80	3%	6
A4. Improve in-vehicle security	2.596	7.12	7%	3	4.302	8.24	18%	2
A5. Improve security at the station	2.027	5.66	4%	5	2.337	4.83	3%	4
A6. Improve road safety	1.799	5.02	3%	6	1.662	3.33	1%	9
A7. Increase the number of places I can accede	1.043	2.80	1%	11	0.192	0.31	0%	12
A8. Increase the number of schedules I can travel	0.000	–	0%	13	0.000	–	0%	13
A9. Improve the frequency of the service	1.562	4.34	2%	9	1.892	3.85	2%	7
A10. Reduce the fare	1.453	3.99	2%	10	− 0.004	− 0.01	0%	14
A11. Reduce pollution	3.213	8.24	12%	2	2.328	4.81	3%	5
A12. Improve reliability in arrival time	1.787	4.97	3%	7	1.186	2.28	1%	11
A13. Improve the neighborhood aesthetic	− 0.031	− 0.07	0%	14	1.213	2.33	1%	10
A14. Improve the quality of life	− 1.033	− 1.84	0%	15	− 1.613	− 1.47	0%	16
A15. Nothing	0.274	0.66	1%	12	1.683	3.35	1%	8
A16. Other	–	–	–	–	− 0.227	− 0.33	0%	15
Panel (std. dev.)	1.378	6.70	–	–	0.757	2.72	–	–
Log-Likelihood		− 2133.0				− 1475.3		
Adjusted Rho2		0.169				0.342		

**Fig. 3:**
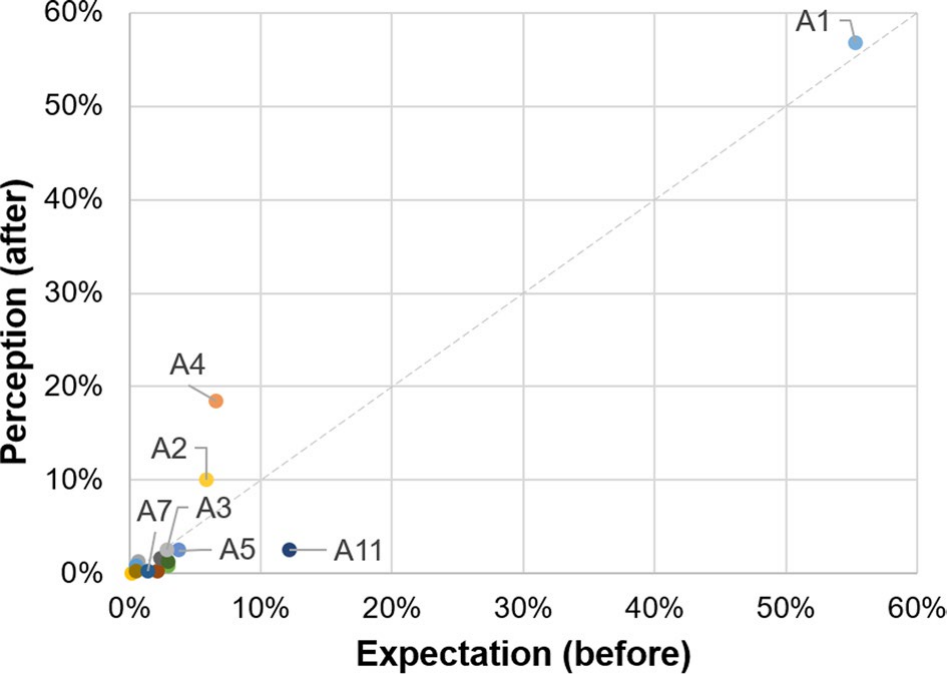
Comparison of probabilities in aggregated models

In contrast, reduction in pollution levels (A11) is the attribute that lost the most ground, moving from second-ranked before implementation to fifth after, suggesting that the users did not perceive relevant improvements in air quality. This may be because the study population is more concerned with costs and occupation than air pollution. It is important to underscore that this population is the poorest and the settlements are in many cases self-built. Fare reductions (A10) did not meet expectations either, even though the parameter is not significant. This could be due to the TransMiCable fare being higher than the cost of paratransit services that were common in the zone, although it is integrated with the city’s public transport system. Also, reductions in travel time (A1), comfort (A2), in-vehicle security (A4), and pollution reduction (A11) dominate the perceptions and expectations. These four attributes can be identified as the highest-ranked by users, but there is no clear difference in the classification of other alternatives, as seen in Fig. 3.

Most parameters are significant at the 5% confidence level, so the statistical difference of the estimates is accepted.

### Second group of models estimation

In this section, we present the results of the estimation of models including the covariates sex, household income, and occupation (i.e., if the respondent is occupied in work or study). An additional interaction was evaluated at the follow-up, considering if the individual had used the TransMiCable at least once (Table 3). This model allows the identification of differences in expectations and perceptions by these attributes. Estimates suggest that expectations vary mainly by occupation rather than by sex or income.

**Table 3:** Models with interactions

Parameter	Attribute	Expectation		Perception	
		Estimate	t-test	Estimate	t-test
ASC	A1. Reduce travel time	4.483	8.56	3.952	5.41
	A2. Improve comfort	2.038	4.85	2.236	2.86
	A3. Improve reliability in waiting time	1.277	2.94	3.038	5.34
	A4. Improve in-vehicle security	2.391	5.63	2.654	3.87
	A5. Improve security at the station	1.583	3.71	− 0.034	− 0.03
	A6. Improve road safety	1.493	2.79	1.714	3.36
	A7. Increase the number of places I can accede	0.606	1.28	0.205	0.33
	A8. Increase the number of schedules I can travel	0.000	-	0.000	–
	A9. Improve the frequency of the service	1.146	2.61	1.951	3.87
	A10. Reduce the fare	1.501	4.05	− 0.008	− 0.01
	A11. Reduce pollution	2.980	6.82	2.407	4.84
	A12. Improve reliability in arrival time	1.940	3.86	1.561	2.75
	A13. Improve the neighborhood aesthetic	− 0.936	-1.34	1.251	2.35
	A14. Improve the quality of life	− 1.006	-1.78	− 1.622	− 1.47
	A15. Nothing	− 1.675	-1.57	1.735	3.39
	A16. Other	–	−	− 0.236	− 0.34
Interaction female	A1. Reduce travel time	–	−	− 0.635	− 2.06
	A2. Improve comfort	–	−	− 0.834	− 2.80
	A3. Improve reliability in waiting time	–	−	− 0.904	− 2.32
	A6. Improve road safety	− 0.602	− 1.60	–	–
Interaction income < 250 USD/month	A1. Reduce travel time	− 0.528	− 1.92	–	–
	A2. Improve comfort	–	–	0.837	3.06
	A4. Improve in-vehicle security	–	–	0.462	1.84
	A12. Improve reliability in arrival time	− 1.049	− 2.74	–	–
	A13. Improve the neighborhood aesthetic	–	-	− 0.859	− 1.47
	A15. Nothing	2.495	2.33	–	–
Interaction occupied	A1. Reduce travel time	1.129	2.95	–	–
	A2. Improve comfort	0.901	2.21	–	–
	A3. Improve reliability in waiting time	0.994	2.21	–	–
	A4. Improve in-vehicle security	0.505	1.28	–	–
	A5. Improve security at the station	0.909	2.13	0.621	1.60
	A6. Improve road safety	1.315	2.83	−	–
	A7. Increase the number of places I can accede	0.885	1.72	−	–
	A9. Improve the frequency of the service	0.840	1.81	−	–
	A11. Reduce pollution	0.575	1.51	−	–
	A12. Improve reliability in arrival time	0.772	1.67	−	–
	A13. Improve the neighborhood aesthetic	1.560	2.02	−	–
Interaction has used TransMiCable	A1. Reduce travel time	–	–	2.571	4.70
	A2. Improve comfort	–	–	1.919	3.19
	A4. Improve in-vehicle security	–	–	1.826	3.44
	A5. Improve security at the station	–	–	2.205	2.01
	Panel (std. dev.)	1.407	6.78	1.013	4.08
	Log-Likelihood	− 2110.5		− 1440.9	
	Adjusted Rho2	0.172		0.353	

Perceptions after the cable car implementation are mainly associated with the use of the service. Compared to the expectations model, fit indices improvements suggest that for perceptions, the inclusion of the interaction term defined by cable users allows capturing more variability of the choices reported, which could not be measured at the expectations stage considering sex, income, and occupation alone. Similar to the first group of models, the standard deviation of the error component associated with the panel effect is highly significant, so the repeated responses of an individual are correlated.

Before the inauguration of the TransMiCable, occupied individuals expected more benefits in most of the attributes, as inferred from the positive sign of the coefficients associated. Nevertheless, after implementing the cable car, these expectations were not met for the segment, as only the interaction of the variable in the utility function of alternative A5 (improvements in security at the station) was significant at the 15% confidence level.

At the follow-up, TransMiCable perceived benefits of travel time (A1), comfort (A2), in-vehicle security (A3), and station security (A4) were significant at the 95% confidence level. TransMiCable users experienced positive changes on these attributes after the implementation. Females seem to perceive fewer benefits in terms of travel time, comfort, reliability of waiting time, and road safety. However, the magnitude of the obtained parameters in Table 3 suggests that the links with the use of the cable car are stronger.

**Fig. 4:**
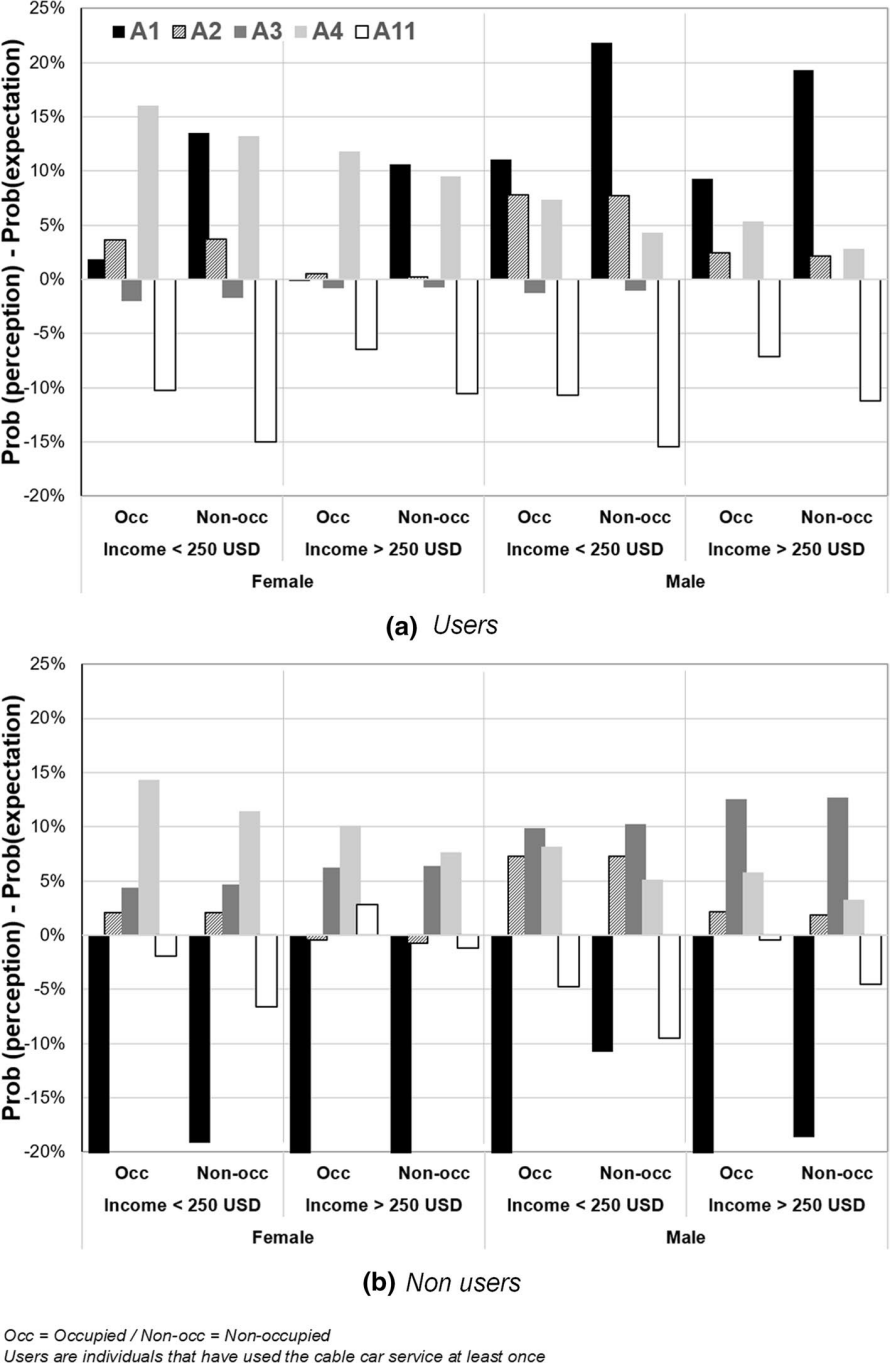
The difference in the probability of ex-post perceptions and ex-ante expectations of users and non-users of the TransMiCable by sub-groups

When comparing expectations versus perceptions, differences are primarily related to the usage of the cable car. Figure 4 presents the net difference in the probability of perceptions and expectations for the five attributes that varied the most for non-users and users of the cable, respectively. In this case, we refer as users to those individuals that have used the cable car service at least once, while non-users never used it. Even though the number of non-users is smaller, there are statistically significant differences in the perceptions of both groups regarding travel time savings, security, and comfort improvements (see Table 3).

For non-users, perceptions of travel time savings (A1) are considerably lower than their expectations, especially among females. In contrast, users’ perceptions of travel time reductions (A1) are much higher, especially for males. Comfort improvements (A2) are valued similarly by users and non-users, but the perceptions are slightly higher for low-income males. Expectations and perceptions of the reliability of waiting time (A3) are similar for users, but non-users perceptions are higher, particularly for males. In the case of in-vehicle security (A4), perceptions exceed the expectations for all users, but most for females. Lastly, TransMiCable users perceive fewer benefits to air pollution (A11) than expected; this aspect does not meet non-users expectations either.

Security at the station (A5) also resulted in lower perceptions than expected for all segments of users, while road safety (A6) did not meet males’ expectations. Also, non-occupied persons perceive fewer cost benefits (A10). However, these attributes are classified in the middle and lower rank positions, so their differences are not as significant as those included in the first four positions (A1, A2, A4, A11), which account for most of the perceived benefits.

## Policy implications and recommendations

The implementation of public transport infrastructure represents a complex challenge for policymakers, especially when it relates to new services that might not be familiar to the potential users. Moreover, the success and acceptance of a project are related to user expectations and perceptions, which vary according to socioeconomic conditions. Hence, the same infrastructure might generate diverse perceived outcomes considering the context in which it is implemented.

In particular, the social and economic conditions of Latin America and developing countries, in general, require an integrated urban transport planning approach that prioritizes investments considering the actual needs and expectations of citizens. To that end, a coordinated effort to involve the community and institutions at different levels through financial instruments, political support, and technical assistance is needed (Hidalgo and Huizenga 2013; Hull 2008). This can be done by developing social participation mechanisms, such as the inclusion of perception analysis in the planning process, and before-after comparisons that help to identify specific project outcomes and fulfillment of goals (Mackett and Edwards 1998).

With this in mind, the results from our research in the case of the TransMiCable provide some guidelines for decision-makers working on new alternative public transport investments in vulnerable zones with low accessibility and transport disadvantages. Together with travel time savings, comfort, reliability, pollution reduction, and security are the most expected benefits of the project. Another critical element to consider is that expectations and perceptions of benefits vary according to socioeconomic characteristics, so population segments perceive infrastructure impacts differently.

The identification of perceptions should be included in communication programs that concentrate on the most relevant attributes perceived by specific user segments. Also, perception modeling is an alternative to model user preferences assessing the service elements that require interventions to improve the quality of service. This is in line with the recommendations of some authors that advocate better integration of social outcomes within public transport policies at the strategic and operational levels (Lucas 2012).

## Conclusions

This paper aimed to evaluate and understand the expectations and perceptions of residents in the area of influence of a new cable car system on the southern periphery of Bogotá. The study area is characterized by (originally) informal settlements, low income, and low accessibility. We designed and issued a panel survey to residents in the catchment area before and after the project was implemented. We asked respondents to rank a list of attributes that they identified as potentially beneficial to them with implementing the cable car. The survey results provide significant insight into the potential for acceptance of similar projects that could be considered to address mobility issues in vulnerable and peripheral urban areas. With this goal, we developed a methodology based on the estimation of two sets of discrete choice models to analyze the attributes that the users expected would improve the most with the cable car, compared with their perceptions after the new system began operation. Models account for perception heterogeneity by incorporating covariate attributes such as sex, household income, occupation, and reported use of the cable car.

Results suggest that travel time saving was the principal expected benefit. Currently, the project meets this expectation. Other relevant anticipated benefits included improvements in travel comfort, in-vehicle security, and reduction of pollution levels. The project also meets these expectations, except for the case of pollution, where users perceived fewer benefits after the cable implementation. Expectations differed according to some characteristics of the population, especially occupation. Regarding perceptions ex-post, heterogeneity is mostly explained by the use of the cable car.

The study also provides information regarding user expectations and perceptions of new public transport investments in the Latin American context. All stakeholders can be informed and persuaded regarding the benefits of this type of project as they engage in the planning process. The above is a crucial issue because cable cars are a new public transport service in the city. This research provides the initial steps towards a more integrative planning process in vulnerable and peripheral urban areas in developing cities, where a patronizing view like ‘implement and surprise’ is the usual practice. Therefore, results are relevant for operators and policymakers to prioritize the design, planning, and operation of public transport services to meet users’ needs and requirements.

Some research limitations that could be addressed in future research include using a bigger sample with more variability. The TransMiCable is located in a zone with a specific context of vulnerability, so further evaluations should be expanded to users with different characteristics. Because the survey instrument did not provide specificity concerning the attributes to rank, the results of the attribute weighting (i.e., pollution) cannot be associated with expected savings regarding a particular trip or those related to the perception of perceived changes in the study area. Therefore, we suggest including more details in the attribute description to be ranked in future assessments. Also, users’ perceptions could be compared to those of policymakers and urban planners to identify differences in their evaluation of attributes. Finally, incorporating latent variable modeling might provide a robust tool to better understand individuals’ cognitive processes by accounting for attitudes and perceptions in the form of latent variables. Although we can obtain information about perception attributes, we decided not to include them because they may vary over time and be highly impacted by a shock or change of this magnitude (e.g., introducing a new transport mode). Jensen et al. (2013) suggest that attitudes may be stable after testing a given alternative. However, their models also suggest that preferences may change, which may also extend to perceptions. Considering the above, we decided to focus on the changes reported according to the ranking itself. The study of the change in perception attributes is also left for further research.
